# Prefrontal cortex interneurons display dynamic sex-specific stress-induced transcriptomes

**DOI:** 10.1038/s41398-019-0642-z

**Published:** 2019-11-11

**Authors:** Matthew J. Girgenti, Eric S. Wohleb, Sameet Mehta, Sriparna Ghosal, Manoela V. Fogaca, Ronald S. Duman

**Affiliations:** 1 Department of Psychiatry, Yale University School of Medicine, 34 Park Street, New Haven, CT 06520 USA; 2 Department of Pharmacology and Systems Physiology, University of Cincinnati College of Medicine, 2120 East Galbraith Road, Cincinnati, OH 45237 USA; 3 Yale Center for Genome Analysis, Yale University School of Medicine, New Haven, CT, 06520 USA

**Keywords:** Molecular neuroscience, Depression

## Abstract

γ-aminobutyric acid (GABA) inhibitory interneurons play a key role in efferent and afferent control of principle neuron activity in the prefrontal cortex (PFC), thereby regulating signal integrity of cognitive and behavioral processes. Recent evidence suggests that specific subtypes of interneurons in the PFC mediate stress-induced depressive-like behaviors. Abnormalities of GABA interneurons, particularly the somatostatin (human, SST; mouse, Sst) subtype, have been reported in postmortem brains of depressed subjects and include sex differences that could explain the increased incidence of depression in women. Here, we analyze the transcriptional profiles and the effects of chronic stress in males vs. females on GABA interneuron subtypes in the PFC. Using *Sst-* and *Parvalbumin*-fluorescence tagged reporter mice and fluorescence-activated cell sorting (FACS) combined with RNA sequencing, we identify distinct transcriptome profiles for these interneuron subtypes in the medial PFC. Based on evidence that SST interneurons are altered in depression, we then determined the effects of chronic stress on this interneuron subtype. Chronic stress causes significant dysregulation of several key pathways, including sex-specific differences in the Sst interneuron profiles. The transcriptional pathways altered by chronic stress in males overlap with enriched pathways in non-stressed females. These changes occurred predominantly in decreased expression of elongation initiation factor 2 (EIF2) signaling, suggesting that dysfunction of the translational machinery of SST interneurons could be critical to the development of depressive-like behaviors in males. In addition, SST interneurons from females exposed to chronic stress show dysregulation of different, growth factor signaling pathways.

## Introduction

γ-aminobutyric acid (GABA) inhibitory interneurons modulate the afferents to dendritic fields of excitatory pyramidal neurons as well as the soma and efferent activity, enhancing the signal to noise ratio and thereby providing critical control of cognition, working memory, mood, emotion, and other complex behaviors^1,2^. Interneurons can be classified based on morphology and on expression of calcium-binding proteins and neuropeptides^3^, which contribute to their neurophysiological properties and their role in the compartmentalized microcircuitry in the cortex^4^.

Clinical and preclinical studies report deficits in specific interneuron subsets that are thought to contribute to the pathophysiology of major depressive disorder (MDD)^5–7^ and schizophrenia^8^, and in the response to stress^9^. Reduced cortical GABA levels^10,11^ and alterations in markers of specific subtypes of interneuron^12,13^ have been reported in postmortem brains of MDD subjects and in animal models of stress^14^. Two of the major interneuron subtypes are characterized by expression of the neuropeptide somatostatin (human, SST; mouse, Sst) (30%), which target dendrite terminals, or the calcium-binding protein parvalbumin (human PVALB; mouse, Pvalb) (40%), which target the somata of principle neurons. MDD patients show a significant reduction of SST-expressing interneurons^12,15–17^. Preclinical studies also show dysregulation of Sst-expressing interneurons after exposure to chronic stress^14,18–21^, and *Sst* deletion mutant mice exhibit behavioral and molecular features of MDD^22^. Cortical Sst interneurons in male mice also display significantly more differentially expressed genes (DEGs) compared to pyramidal neurons after chronic stress^23^. Together, these results suggest that stress impairs interneuron function in the medial PFC, especially in SST-expressing interneurons, and this contributes to principle neuron dysfunction and the development of anxiety- and depressive-like behaviors^24,25^.

MDD is markedly more prevalent in women than in men^26–31^. Women manifest different symptomology^32–34^, treatment responses^30^, and comorbidities^35^. The mechanisms underlying this sexual dimorphism are unclear. Postmortem transcriptome studies report that female MDD subjects have greater reductions in SST immunolabeling in anterior cingulate cortex than male MDD subjects^15,16^ and show reductions in amygdala SST, which are not observed in males^17^. However, the molecular and cellular mechanisms underlying these sex differences have not been determined.

Here we used cell type specific RNA sequencing (RNA-seq) to identify the molecular differences between male and female mice in two major cortical interneuron subtypes, Sst and Pvalb, and examine the sex-specific effects of chronic unpredictable stress (CUS) on the Sst subtype transcriptome. We find sex-dependent differences between the transcriptomes of SST and Pvalb interneurons, as well as sex-specific differential effects of CUS. Some of these effects parallel those observed in a recent human postmortem MDD gene expression data^36^. These results are the first to show sexual dimorphism of the transcriptome in a specific interneuron subtype under basal conditions and in response to stress exposure, effects that could contribute to sex-specific behavior differences in stress and depression.

## Materials and methods

### Animals

Male and female transgenic mice and wild-type littermates were obtained from in-house breeders. *Sst Cre* recombinase (*Sst*^CRE^) (#013044) and *Pvalb* Cre recombinase (Pvalb^CRE^) (#008069) mice were obtained from Jackson Laboratories and crossed with Ai9(RCL-tdTomato) mice that are Cre recombinase dependent (#007909), yielding *Sst*^*tdT*^ or *Pvalb*^*tdT*^ reporter mice. Behavioral studies utilized wild-type littermates from *Sst*^*tdT*^ cohorts; subsets of wild-type C57BL/6 mice were included to confirm behavioral responses. All behavioral and transcriptomic studies used animals heterozygous for *Sst*^*tdT*^ or *Pvalb*^*tdT*^. All studies were performed with mice 6–12 weeks old. Mice were housed in 11.5” × 7.5” × 6” polypropylene cages under a 12 h light-dark cycle with ad libitum access to water and chow. Animal use and procedures were in accordance with the National Institutes of Health guidelines and were approved by the Yale University Animal Care and Use Committee.

### Interneuron FACS and mRNA isolation

To isolate interneurons with tdTomato expression, PFC was rapidly dissected placed in digestion buffer (PBS/10% Papain). Following 20 min incubation at 37 °C, digestion was stopped with FACS buffer (PBS/1% BSA), followed by gentle dissociation with a glass Dounce homogenizer (Kontes Glass Company, Vineland, NJ). Single-cell solutions were spun down at 1000 rpm for 5 min, re-constituted in 600 μl of FACS buffer, and filtered (40 μm) into flow tube. Samples were sorted on a FACS Aria (Yale Immune Monitoring Core Facility) into lysis buffer; average yield was ~150,000 cells/sample. RNA was obtained with the Norgen Single Cell RNA Purification Kit (#51800; Norgen Biotek Corp., Thorold, Ontario, Canada).

### RNA-sequencing

100 ng of total mRNA was processed using the Pico RiboGone-Mammalian kit (TakaraBio, Kyoto, Japan) before cDNA synthesis. RNA-sequencing was performed on an Illumina HiSeq2500 using 75-bp, paired-end sequencing, at 35–40 million reads per sample.

### Real-time qPCR

RNA from FAC sorted cells was collected using an RNAqueous- micro kit based on the manufacturer’s protocol (Thermo Fisher, Waltham, MA). RNA (100 ng) was reverse transcribed to cDNA using the Superscript VILO cDNA synthesis kit (Thermo Fisher, Waltham, MA). Primers were designed with Primer3. cDNA was amplified on a CFX96 detection system (BioRad) by real-time PCR and normalized based on reference gene expression (GAPDH). Data were analyzed with the comparative threshold cycle method.

### Immunohistology

Brains were collected from mice after transcardiac perfusion with sterile PBS and 4% paraformaldehyde (PFA). Brains were post-fixed in 4% PFA for 24 h and incubated in 30% sucrose for an additional 24 h. Fixed brains were frozen and sectioned at 50 µm using a Microm HM550 cryostat. Free-floating sections were washed, then blocked for 1 h at room temperature. Sections were washed, then incubated with primary antibodies (1:1000 dilution): mouse anti-somatostatin (Millipore; MAB354) or rat anti-parvalbumin (Millipore; MAB1572) overnight at 4 °C. Sections were then washed and incubated with conjugated secondary antibody (1:1000 dilution) overnight at 4 °C. Brain sections were mounted on microscope slides and cover-slipped using Fluoromount-G and cell counts were performed by a condition blinded experimenter.

### Quantitative Immunofluorescence

For quantification of *Sst*^*tdT*^ or *Pvalb*^*tdT*^ interneurons, 2–3 adjacent brain slices containing the prelimbic and infralimbic subregions of the prefrontal cortex (Bregma 1.95–1.55) were selected for immunohistology. Bilateral images spanning all lamina were obtained in the prelimibic and infralimbic subregions. Immunofluorescence was visualized using an Olympus BX61WI confocal microscope with a ×20 objective (NA: FN26.5) (Tokyo, Japan). Images were captured with Fluoview (FV1000) and Hamamatsu high-resolution digital camera (ORCA-ER; Hamamatsu City, Japan). Manual cell counts were quantified by a blinded experimenter. Counts were summarized in each lamina based on distance from the midline, and averaged in each subregion, and the average across samples was used for statistical analyses (*n* = 4–6).

### Bioinformatics

FASTQ files were trimmed and assessed for quality before being annotated with the mouse genome (mm9) using TopHat2^37^. No bases were accepted below a quality of Q30. If the read was trimmed below the length of 45 bp, then the read was discarded. The reads were aligned to the mm10 reference genome using HISAT2 aligner^38^. The gene level and transcript level counts were obtained using stringTie^39^. The differential gene expression analysis was done using DESeq2^40^ with its default settings for scaling and transforming the data. The count data were used for differential gene expression analysis, all the visualization was done using FPKM values calculated per sample per gene. Genes were deemed significantly expressed if FKPM > 0.4. Samples that varied significantly from the group mean (>2 standard deviations) were eliminated from further results. Results were visualized using R (The Comprehensive R Archive Network. Cran.R-Project.org) and cummerbund^41^ (all codes used are publically available). Batch effects were removed using ComBat script. DEGs were assessed using Fisher’s exact test corrected for multiple comparisons (Benjamini-Hochberg, FDR of 0.05).Enrichment of gene ontology, network building, upstream and node gene identification were performed using Ingenuity Pathway Analysis (IPA) (Qiagen Inc., https://www.qiagenbioinformatics.com/products/ingenuity-pathway-analysis). Activation/inhibition states of the top enriched canonical pathways were examined using IPA’s activation *Z*-score tool. IPA’s network generation algorithm was used to identify the highly interconnected networks of the top ranked genes. All raw and processed sequencing data are available on GEO, accession number GSE138670. To identify correlations between the findings of the current study in mice with the results from a published study in humans, we reanalyzed and compared the male and female transcriptomic datasets^36^ for differential gene expression overlap between males with MDD and control females in area 25 (ventromedial prefrontal cortex). Area 25 was chosen as it is the most homologous human region to mPFC. Datasets were processed using IPA to identify overlapping transcripts between the cohorts. The overlapping transcripts were further processed for pathway identification and enrichment.

### Chronic unpredictable stress (CUS)

In the present study mice underwent 14 days of CUS exposure, as previously described;^42^ CUS included random exposure to light overnight, cage tilt, wet bedding, rat odor, white noise, food deprivation, water deprivation, light off during day, isolation, restraint, and strobe light. CUS exposure generally occurred over 4–6 h intervals in the morning and evening. This is a well-studied stress paradigm causing robust neurobiological changes that contribute to development of depressive-like behaviors^43,44^.

### Behavioral testing

#### Anxiety-like behavior

For open-field activity, mice were placed in a Plexiglas test apparatus (40 × 40 × 25 cm) and recorded for 15 min. Activity in the open-field was analyzed automatically (ANY-maze, Stoelting Company).

#### Forced Swim Test (FST)

FST was conducted as previously described^44^. Mice were placed for 10 min in a clear cylinder filled with water (24 ± 1 C, 18 cm depth). Sessions were video-recorded and scored for total immobility time. Time immobile during the 2–6 min block is reported.

### Statistical analysis

Data were analyzed with IBM SPSS Statistics and GraphPad Prism. Significant main effects and interactions were determined using one- (sex, treatment), or two- (sex × treatment) way ANOVA. Grouped analyses were completed without repeated measures and differences between group means were evaluated with Fischer’s least significant differences (LSD) test. Significant post-hoc group differences are only reported if protected by significant main effects or interactions with ANOVA. To determine significance between overlapping transcript lists we employed exact hypergeometric probability calculating whether the number of overlapping genes drawn from two independent lists is greater than expected by chance. Quantitative PCR data were analyzed using non-parametric Mann–Whitney *U*-test.

## Results

### Transcriptomic profiling of male vs. female Sst interneurons reveals unique profiles

**Fig. 1:**
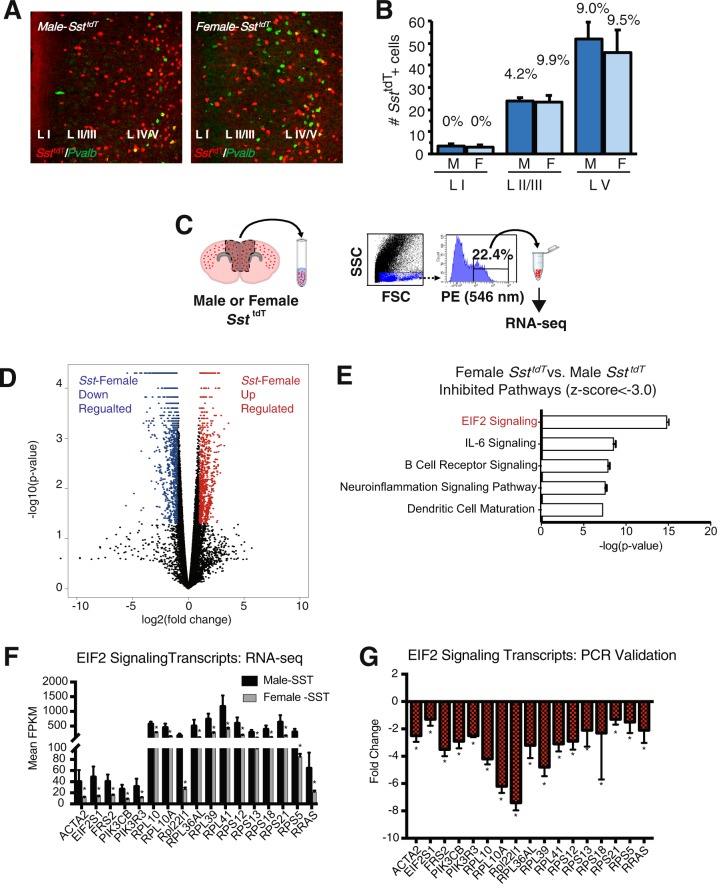
Transcriptomic analysis reveals differential expression in male and female *Sst*^*tdT*^ interneurons. **a** Representative confocal images of *Sst*^*tdT*^ mice with co-labeling for Pvalb. **b** Quantification of average number of *Sst*^*tdT*^ interneurons in each lamina of the medial PFC, percent represents proportion of *Sst*^*tdT*^ interneurons with co-expression of alternate marker. Here we show that 0–9.9% of *Sst*^*tdT*^ co-expressed Pvalb (*n* = 3/group; error bars indicate ± S.E.M.). **c** Schematic of procedure for FAC sorting interneuron subtypes followed by RNA-seq. **d** Volcano plot of genes differentially regulated between male and female *Sst*^*tdT*^ interneurons. Red dots indicate upregulated transcripts and blue dots represent downregulated transcripts. There are 1995 DEGs, *n* = 4–7 (FDR < 0.05). **e** Top five most significantly inhibited pathways (mostly downregulated) in female *Sst*^*tdT*^ interneurons vs. males. EIF2 signaling was the top inhibited pathway in females. **f** Mean FPKM of EIF2 signaling transcripts significantly lower in female *Sst*^*tdT*^ interneurons error bars indicate ± S.E.M. (FDR < 0.05). **g** Secondary confirmation of EIF2 signaling transcript expression levels in baseline male and female *Sst*^*tdT*^ interneurons by qPCR error bars indicate ± S.E.M. of log corrected Ct values (*n* = 3, Mann–Whitney*-U*, *p* < 0.01,)

Recent postmortem studies of MDD reveal whole-tissue transcriptomic differences in male and female subjects^36,45^. To examine possible sex differences in Sst interneurons, we characterize the transcriptome of GABA interneuron subtypes using transgenic mice with fluorescent tagged cells. Examination of the cell distribution shows the expected pattern of *Sst*^*tdT*^ positive cells across all layers of mPFC, with no significant differences in laminar distribution of Sst cells between males and females (Fig. 1a). To examine the integrity of the *Sst*^*tdT*^ mouse line for each interneuron subtype, we determined the overlap of *Sst*^*tdT*^ and Pvalb immunopositive cells and demonstrate very low (<10%) overlap between *Sst*^*tdT*^ interneurons with Pvalb positive cells (Fig. 1b). Cell counts show the lowest number of *Sst*^*tdT*^ positive cells in layer I with the maximum number observed in layer V (Fig. 1b). To confirm specificity of the td-Tomato tag we performed immunohistochemistry for endogenous Sst in mPFC sections of male *Sst*^*tdT*^ mice to confirm overlap (Fig. S1A).

Next we used FACS to selectively isolate *Sst*^*tdT*^ tagged cells from the mPFC of males and females, RNA was isolated, and RNA-seq was conducted (Fig. 1c). Overall, there were 12,585 co-expressed genes in male and female *Sst*^*tdT*^ tagged interneurons (Fig. 1d), with a high degree of correlation (*R*^2^ = 0.78, *Pearson’s* correlation) (Fig. S1B, Table S1). We found 1995 DEGs (FDR < 0.05), with 1461 genes downregulated (blue) and 534 upregulated (red) in females compared to males (Fig. 1d). Altered pathways were identified using IPA (all pathways are rank ordered by p-value). Notably, EIF2 signaling was the top inhibited pathway in females (Fig. 1e, f). A previous study of Sst interneurons conducted in males only reported a reduction in EIF2 signaling genes in response to repeated stress^23^. We confirmed the base line differences of seventeen genes in the EIF2 signaling significantly lower in females versus males by qPCR analysis (Fig. 1g, *p* < 0.01).

### Male and female *Pvalb*^*tdT*^ interneurons exhibit similar transcriptomic profiles

**Fig. 2:**
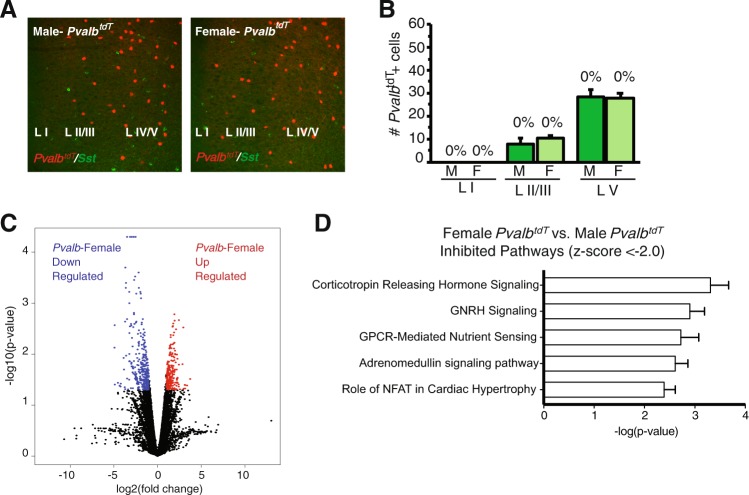
Transcriptomic analysis reveals few transcriptomic differences between male and female *Pvalb*^*tdT*^ interneurons. **a** Representative confocal images of *Pvalb*^*tdT*^ mice with co-labeling for Sst. **b** Quantification of average number of *Pvalb*^*tdT*^ interneurons in each lamina of the mPFC; percent represents proportion of *Pvalb*^*tdT*^ interneurons with co-expression of Sst. Here we show that 0% of *Pvalb*^*tdT*^ interneurons co-expressed Sst, error bars indicate ± S.E.M. (*n* = 3/group). **c** Volcano plots of differential gene expression between female and male *Pvalb*^*tdT*^ interneurons. Red dots indicate upregulated transcripts and blue dots represent downregulated transcripts in the female *Pvalb*^*tdT*^ interneurons. There are 112 DEGs (FDR < 0.05), *n* = 2–7 in *Pvalb*^*tdT*^ males vs. females. **d** Bar graph shows top 5 pathways for DEGs between male and female *Pvalb*^*tdT*^ that were inhibited and rank ordered by *z*-score

We examined *Pvalb*^*tdT*^ expression across the mPFC of male and female mice and found no significant difference in the number of *Pvalb*^*tdT*^ labeld cells between males and females (Fig. 2a). To examine the specificity of the *Pvalb*^*tdT*^ mouse line, we determined the overlap of immunopositive cells and found very low or no overlap of *Pvalb*^*tdT*^ and Sst neuropeptide immunolabeled cells (Fig. 2a, b). We detected few *Pvalb*^*tdT*^ positive cells in layer I and found layer V contained the most Pvalb positive cells. Unlike the profiles from *Sst*^*tdT*^ mice, interneurons from male and female *Pvalb*^*tdT*^ mice had relatively similar transcriptomic profiles. Male and female *Pvalb*^*tdT*^ interneurons co-expressed 12,749 genes. Of these, there were 112 DEGs. Further analysis revealed several pathways were decreased in females relative to males (Fig. 2d, Table S1) but notably not the EIF2 pathway, indicating this difference is selective to Sst interneurons.

### Interneuron subtype-specific transcriptional profiling in the PFC

Next, we compared the molecular profiles of the PFC from naive, nonstressed *Sst*^*tdT*^ and *Pvalb*^*tdT*^ mice (Fig. S2a). We found enrichment of *Sst* transcript in the *Sst*^*tdT*^ interneurons and enrichment of *Pvalb* transcript in the *Pvalb*^*tdT*^ cells, indicating relatively pure sorting of each cell type (Fig. S2b). The transcriptomes of *Sst*^*tdT*^ and *Pvalb*^*tdT*^ interneurons showed broad overlap between cell types, but also significant differences. Of the 12,622 co-expressed genes, 1481 were DEGs (Fig. S2C); 156 genes were expressed in *Pvalb*^*tdT*^ interneurons exclusively, while 228 were expressed only in *Sst*^*tdT*^ interneurons (FDR < 0.05). We identified different pathways enriched in *Pvalb*^*tdT*^ interneurons (Fig. S2D) indicating interneuron subtypes have transcript expression pattern differences.

### Chronic stress exposure alters multiple transcriptome signaling pathways in male *Sst*^*tdT*^ interneurons

**Fig. 3:**
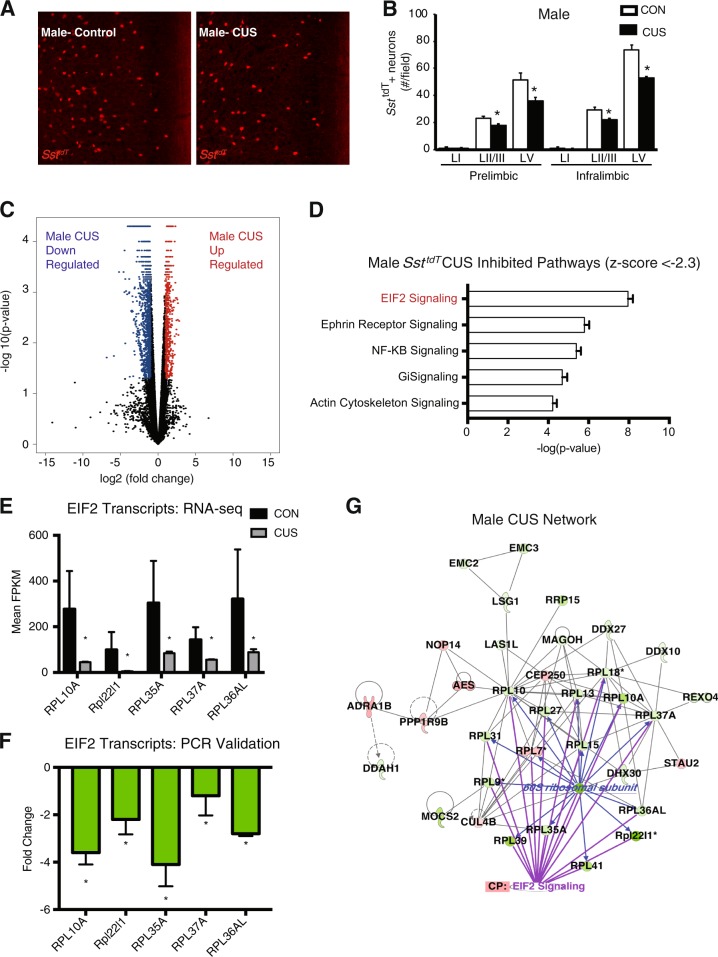
CUS alters *Sst*^*tdT*^ interneuron number and transcription patterns in the medial PFC of male mice. Male *Sst*^*tdT*^ mice were exposed to 14 days of CUS and 2–4 h after the final stressor mice were perfused and brains collected for histology. **a** Representative images of *Sst*^*tdT*^ interneurons in the infralimbic subregion of the medial PFC of CUS exposed and control male mice. **b** Average number of *Sst*^*tdT*^ interneurons in each layer of prelimbic and infralimbic medial PFC are shown; bars are the mean ± S.E.M. (*n* = 4–7/ group). Means that are significantly different than respective control group based on ANOVA are noted: *p *<* 0.05). **c** Volcano plot of genes differentially regulated by CUS, *n* = 3–7 (FDR < 0.05). Red dots indicate upregulated and blue dots represent downregulated transcripts (FDR < 0.05). There were 1581 genes differentially regulated by CUS. **d** Top 5 signifcantly inhibited pathways in CUS male *Sst*^*tdT*^ interneurons. EIF2 signaling is the most inhibited pathway (most number of downregulated transcripts). **e** Mean FPKMs of EIF2 signaling transcripts in the CUS network; error bars indicate ± S.E.M. (FDR < 0.05). **f** Secondary confirmation of EIF2 signaling transcripts in the *Sst*^*tdT*^ male CUS network by qPCR; error bars indicate ± S.E.M. of log corrected Ct values (*n* = 3, Mann–Whitney *U*, *p* < 0.01). **g** Ingenuity pathway analysis generated CUS network. This pathway received a score of 35 and has 34 focus molecules with a hub at the 60 s ribosomal subunit. CP indicates the canonical pathway EIF2 signaling and purple lines indicate the genes that are members

Previous studies report altered SST expression is associated with depression and *Sst* gene deletion contributes to depressive-like behaviors in male mice^16,23^. There is less evidence of altered PVALB interneurons in MDD^46^. To further examine the role of Sst interneurons in stress responses, male and female *Sst*^*tdT*^ mice were exposed to 14 days of CUS. Counts revealed CUS reduced the number of *Sst*^*tdT*^ interneurons in layers II/III and V of the prelimbic PFC (main effect of CUS: F_1,27_ = 20.09, *p* < 0.0001) and infralimbic PFC (main effect of CUS: F_1,27_ = 72.11, *p* < 0.0001) of male mice (Fig. 3a, b); this could reflect a reduction of *Sst*^*tdT*^ cell number or decreased *Sst* expression. CUS did not significantly affect the number of *Sst*^*tdT*^ interneurons in layers I–V in the somatosensory cortex, though there was a modest reduction in layer VI (F_1,10 _= 12.9, *p* < 0.005; Fig. S3), indicating selectivity for reductions in PFC. Thus, CUS leads to deficits in Sst interneurons in mPFC, similar to what has been observed in postmortem tissue of depressed subjects. Behavioral analysis showed CUS exposure decreased the time spent in the center of the open field, and increased the latency to enter the center, indicating increased anxiety. In the FST, CUS significantly increased immobility time, a measure of behavioral despair (Fig. S4D, E). There was no significant effect of CUS on distance traveled in the open field (Fig. S4B).

*Sst*^*tdT*^ cells were isolated from PFC of male mice after 14 days of CUS exposure; RNA-Seq was performed on equivalent numbers of purified *Sst*^*tdT*^ interneurons from the PFC. CUS resulted in differential expression of 1581 genes (CUS-DEGs) compared to control non-stressed male mice, with 1083 genes downregulated (blue) and 498 upregulated (red) (FDR < 0.05) (Fig. 3c, Table S1). Notably, the top CUS-inhibited pathway was EIF2 signaling, the same pathway identified to be significantly lower in naive, unstressed females compared to unstressed males (Fig. 3d, e). To validate the expression changes identified by RNAseq we performed quantitative real-time PCR, and confirmed downregulation of all of the EIF2 signaling genes tested, including *Rpl10a*, *Rpl221*, *Rppl35a*, *Rpl37a*, and *Rpl35al*) (*p* < 0.01) (Fig. 3f).

We also performed gene network co-expression analysis to determine if there is an Sst interneuron-specific transcriptional network induced by CUS in males (Fig. 3g). We identified a network centered around the inhibited 60S ribosomal subunit hub gene. The 60S ribosomal subunit makes connections with 15 CUS regulated genes (blue lines). Analysis of connections between EIF2 signaling members with the 60S ribosomal hub reveal 16 overlapping network members (purple lines). Taken together, these results indicate CUS exposure results in transcriptional alterations leading to inhibition of translation in male Sst interneurons.

### CUS exposure results in non-overlapping sex-specific *Sst*^*tdT*^ transcriptional responses

**Fig. 4:**
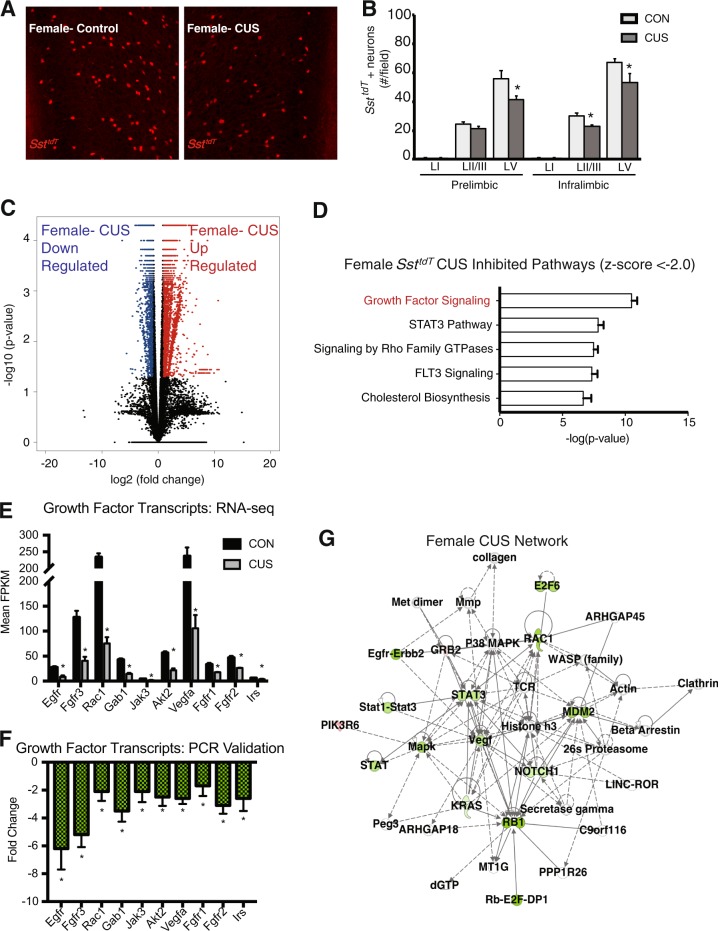
Influence of CUS on female *Sst*^*tdT*^ interneuron transcript expression. **a** Representative images of *Sst*^*tdT*^ interneurons in the infralimbic subregion of the medial PFC of female mice exposed to CUS or left undisturbed as controls. **b** Average number of *Sst*^*tdT*^ interneurons in each layer of prelimbic and infralimbic medial PFC are shown in bar graph. Bars represent the mean ± S.E.M. (*n* = 4–7/ group). Means that are significantly different from respective controls by ANOVA are noted by (*p *<* 0.05). **c** Volcano plots of differential gene expression between *Sst*^*tdT*^ interneurons of female control and CUS exposed. Red dots indicate upregulated transcripts and blue dots represent downregulated transcripts. There are 4642 DEGs (FDR < 0.05), *n* = 3–7 *Sst*^*tdT*^ males and females. **d** Top 5 significantly inhibited pathways in female *Sst*^*tdT*^ interneurons after CUS. The most inhibited (most downregulated genes) was the pancreatic adenocarcinoma signaling pathway comprised mostly of growth factors and growth factor signaling molecules. **e** Mean FPKMs of growth factor signaling transcripts decreased by CUS (FDR < 0.05) in the female CUS network; error bars indicate ± S.E.M. **f** Secondary confirmation of growth factor signaling transcripts in the *Sst*^*tdT*^ female CUS network by qPCR; error bars indicate ± S.E.M. of log corrected Ct values (*n* = 3, Mann–Whitney*-U*, *p* < 0.01). **g** Ingenuity pathway analysis generated a female CUS network centered on growth factors of the adenocarcinoma pathway. This network received a score of 17 and has 9 focus molecules with major hubs at *Vegf* and *Rac1*

We next examined the influence of CUS on female *Sst*^*tdT*^ interneurons. CUS exposure decreased *Sst*^*tdT*^ interneuron numbers in the mPFC, similar to males; there was a significant reduction in *Sst*^*tdT*^ in layer V of prelimbic (F_1,30_ = 4.65, *p* < 0.04) and infralimbic (F_1,30_ = 4.88, *p* < 0.04) cortex of female mice exposed to CUS with a tendency for a decrease in layer II/III of IL (*p* = 0.1) (Fig. 4a, b). Behavioral studies showed exposure of female mice to CUS produced effects similar to males in the open field test with decreased time spent in the center of the open field, and increased latency to enter the center (Figure S4C, D). Effects in the FST in females were also similar to males, with CUS exposure causing a trend for increased in immobility time (Fig. S4E, #*p* = 0.08). There was no significant effect of CUS in females on distance traveled in the open field (Fig. S4B).

Compared to non-stressed females, CUS-exposed females show transcript changes in 4642 genes in *Sst*^*tdT*^ interneurons (female CUS-DEGs), 2185 downregulated (blue) and 2457 upregulated (red) (FDR < 0.05) (Fig. 4c, Table S1). Interestingly, the EIF2 signaling pathway was not effected. Correlation between male and female CUS-DEGs was low (*R*^2^ = 0.302, *Pearson’s* correlation; Fig. S1C, upper panel). There was overlap of 941 genes between the male and female CUS-DEGs (601 up- and 340 downregulated; Fig. S1C, lower panel, Table S2). These data demonstrate CUS has a differential effect on the Sst interneuron transcriptomes of females compared to males.

We identified several CUS-disrupted pathways in female *Sst*^*tdT*^ interneurons, with pancreatic adenocarcinoma (primarily growth factors and refered to as such) being the most significantly altered (37 genes down- and 18 upregulated; Fig. 4d, e). We confirmed the downregulation of the top 10 significant growth factor signaling genes altered by CUS using qPCR (Fig. 4f, *p* < 0.01). IPA identified a growth factor specific network, consisting of several distinct hubs including *Vegf*, *Rac1, Notch1, Stat3*, and *Mdm2* (Fig. 4g). This network is wholly distinct from those identified in the male *Sst*^*tdT*^ CUS-DEGs, demonstrating a fundamental difference between the effects of CUS on male and female Sst interneuron transcriptomes.

### Sex-specific overlap between *Sst*^*tdT*^ interneurons and human postmortem transcriptomes

**Fig. 5:**
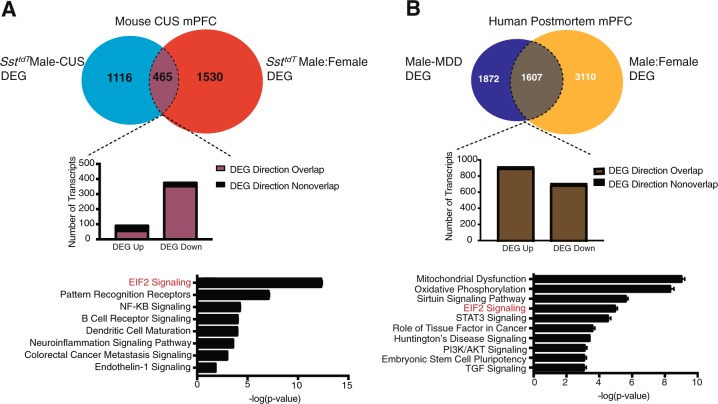
Comparison between mouse *Sst*^*tdT*^ interneuron CUS and human MDD transcriptional patterns. **a** Venn diagrams show overlap between *Sst*^*tdT*^ male CUS DEGs and *Sst*^*tdT*^ control female DEGs. 465 transcripts overlap between the two lists (*p* < 0.0001, exact hypergeometric probability). The bar graph shows the number of transcripts that are up or downregulated and the number that overlap in change and direction. There are 90 genes that were upregulated (55 overlapped and 35 no overlap). There are 375 genes that were downregulated (352 overlapped and 23 did not). Pathway analysis of the 465 overlapping transcripts reveals enrichment for EIF2 signaling (lower panel). **b** Transcript overlap between human males with MDD and control human females. The 1607 overlapping transcripts (*p* < 0.0001, exact hypergeometric probability) were analyzed for pathway enrichment (bottom panel). Middle bar graphs show the overlap in expression change between control females and male MDD subjects. Only 2 transcripts of 908 did not overlap for the upregulated direction and only 3 transcripts of 700 did not overlap in the downregulated direction

We compared *Sst*^*tdT*^ male-female DEGs to *Sst*^*tdT*^ male CUS-DEGs. There was a statistically significant (*p* < 0.0001) 23% overlap (Fig. 5a). Of these 465 genes, 383 differed in the same direction in non-stressed females as in CUS males, while only 82 differed in the opposite direction (Fig. 5a, Table S3). Most were downregulated (375 genes, Fig. 5a, middle panel). When we analyzed the 465 overlapping genes, we found the genes predominantly fell within an inhibited EIF2 signaling pathway (Fig. 5a, lower panel).

To examine the translational relevance of these sex-specific *Sst*^*tdT*^ transcriptional changes to MDD subjects, we used a publicly available transcriptome dataset (GEO, GSE102556) from Broadmann area 25 to identify human correlates to the mouse expression data^36^. We identified 46 enriched pathways between human females and males; the EIF2 signaling pathway was among them (number 13 of 46) (Fig. S5). We next compared DEGs in males with MDD to male-female human DEGs (Table S4). Of the 3479 DEGs in male MDD, 1607 (46%; *p* < 0.0001) overlapped with the male-female DEGs (Fig. 5b, top panel). Most genes were upregulated (908 genes) and only 2 genes from the male-female DEG comparison were expressed in the opposite direction. There were 700 downregulated genes overlapping and only 3 genes did not overlap in direction with the male-female DEG comparison. Pathway analysis of the overlapping genes revealed EIF2 signaling as a top pathways (Fig. 5b, bottom panel). Remarkably, EIF2 signaling is similar in control human females and depressed males (Fig. 5b), similar to our finding that EIF2 signaling in unstressed control female mice is similar to CUS exposed males.

## Discussion

Recent studies have begun to elucidate the transcriptomic architecture of depression in humans, including sex-specific differences^16,36^. These studies have been conducted on bulk tissue limiting the interpretation of dysregulated transcription in specific populations of cells. Here we have used *Sst*^*tdT*^ labeld cells and FACS to isolate a relatively pure population of Sst interneurons and show cell type- and sex-specific transcriptome alterations associated with chronic stress. These cell type-specific changes contribute to the heterogeneity of transcriptomic responses observed in RNA extracted from bulk tissue samples containing hundreds of cell types. This is the first study to combine cell subtype specificity and high throughput RNA-seq to parse transcriptomic differences in stress responses between males and females.

Comparison of male versus female transcriptome differences in *Sst*^*tdT*^ interneurons showed a high level of co-expressed genes, but also a relatively large number of significant DEGs (1995 genes). Most were downregulated in females compared to males. In contrast to the large number of DEGs in *Sst*^*tdT*^ interneurons, there were relatively few sex-specific DEGs in *Pvalb*^*tdT*^ interneurons (112 genes). Further analysis of *Sst*^*tdT*^ interneurons revealed the pathway with the greatest decreased in females compared to males was EIF2 signaling, which plays an integral role in the regulation of protein synthesis. Low levels of EIF2 signaling in females is notable, since a previous report conducted on males only demonstrated that EIF2 signaling was the most downregulated pathway in Sst interneurons exposed to chronic stress^23^, consistent with the results of the current male *Sst*^*tdT*^ CUS results.

Analysis of CUS effects on transcriptome changes in *Sst*^*tdT*^ interneurons shows further sex-specific differential effects. *Sst*^*tdT*^ interneurons from males displayed 1581 DEGs, with the majority, 1083 downregulated, and the most highly downregulated pathway EIF2 signaling. Females exposed to CUS exhibit more transcriptomic changes in *Sst*^*tdT*^ interneurons than males (4642 DEGs), indicating sex-specific responses. While there were baseline differences in EIF2 signaling (decreased in naive, nonstressed females vs. males), there was no further effects of CUS on this pathway. Rather, females exposed to CUS showed transcriptomic alterations in *Sst*^*tdT*^ interneurons not observed in males, most notably growth factor signaling.

These findings provide convergent evidence that EIF2 acts as a signaling pathway after exposure to chronic stress in males, in agreement with a previous study^23^ that found EIF2*-*related genes to be suppressed in stress-exposed Sst interneurons. Disruption of EIF2 signaling and subsequent reductions in protein translation have been implicated in several neurodegenerative disorders^47–49^ as well as rodent models of sociability and anxiety^50^. Further analysis reveals a co-expression network with the 60S ribosomal subunit as a major node with numerous additional RNA-binding proteins involved in translation control, all significantly downregulated. Rapid acting antidepressants, such as ketamine, increase synaptic protein synthesis via the mTORC1 signaling pathway^51^, which would oppose the disruption of translation indicated by the current findings. It remains unknown how aberrant EIF2 signaling in Sst interneurons and the neurobiological effects of stress are mechanistically linked.

We also identify further sex-specific effects in the transcriptome of *Sst*^*tdT*^ interneurons. While EIF2 was the most inhibited pathway in CUS-exposed males, it was significantly lower in unstressed female SST-expressing interneurons. Indeed, male-female *Sst*^*tdT*^ DEGs overlap 42% with *Sst*^*tdT*^ CUS-DEGs in males. Overlap is seen in many pathways beyond EIF2, including inhibition of *Nfkb* and inflammatory signaling. These parallels suggest aspects of the *Sst*^*tdT*^ interneuron transcriptome that are disrupted by stress in males are already lower at baseline in females.

Remarkably, 46% of human male MDD DEGs overlap with human baseline control male-female DEGs. Moreover, EIF2 signaling is lower in control human females, similar to the effect we observed in control female mice. Women are more likely to be diagnosed with single episode or recurrent MDD than men^29,30^ and the results of the current study shed light on a potential mechanism by which SST interneuron transcriptomes respond to stress and how the differences are similar across species.

Decreased EIF2 signaling in females does not appear to effect behavioral measures of depression as similar anxiety- and depression-like behaviors are observed in male and female mice exposed to CUS (Fig. S4). The female animals only perform worse than the males in entries to the center of the open field (Fig. S4D), an indication of anxiety. A major difficulty in these studies is female rodents (mice and rats) display mixed behavioral responses depending on the model, including FST, learned helplessness, and sucrose preference, a measure of anhedonia^52–54^. The overlap between baseline females and CUS males raises important considerations regarding CUS, as well as other preclinical stress models. While human females are more likely to develop depression, it is important to consider female rodents do not always display a similar vulnerable behavioral phenotype to stress compared with male rodents^55–57^. Historically, these studies have been conducted predominantly in males, and some behavioral paradigms are particularly problematic. For example, in the chronic social defeat model, the dominant male used for defeat of subordinate males will not attack female mice, making it very difficult to use this model for studies of females. There have been recent modifications, but social defeat is still not used as a mainstream model for female studies^58,59^. These studies highlight the need for development of additional behavioral models displaying increased vulnerability in female rodents similar to what is observed in humans.

To conclude, we have performed the first study of the sex-specific transcriptomic changes of interneuron subtypes and the effects of chronic stress on these cells. The results have identified a chronic stress co-expression transcriptomic network in Sst interneurons implicating deficits in protein translation as most effected by CUS exposed males. Further, the data demonstrate that unstressed female Sst interneurons are molecularly similar to male CUS exposed Sst interneurons, a finding in general agreement with results of transcriptome studies of human female controls compared with male MDD subjects. Further, in CUS exposed female Sst interneurons, there is disruption in different pathways, notably growth factor signaling, that is not observed in male Sst interneurons. Further investigation should extend this work to assess the transcriptomic profiles of other neuronal cell types with an emphasis on sex-specific differences. It would also be interesting to investigate the influence of antidepressant treatments on sex-specific differences of the Sst interneuron transcriptome. As noted earlier, pathways controlling protein synthesis (i.e., mTORC1 and eEF2 kinase) are downstream of the major targets of rapid acting antidepressants like ketamine, and it is possible these agents could reverse, in part, the stress-induced transcriptomic changes. The results of the current and future studies could provide insights for the development of more effective, and even sex- and cell-specific therapeutic agents for the treatment of MDD.

## Supplementary information

Supplemental Figures

Supplemental Table 1

Supplemental Table 2

Supplemental Table 3

Supplemental Table 4

Supplemental legends
