# Supplementary material for: Prefrontal cortex interneurons display dynamic sex-specific stress-induced transcriptomes
Source: Transl Psychiatry. 2019 Nov 11;9:292. doi: 10.1038/s41398-019-0642-z (PMC6848179; doi:10.1038/s41398-019-0642-z)

Figure S1

A

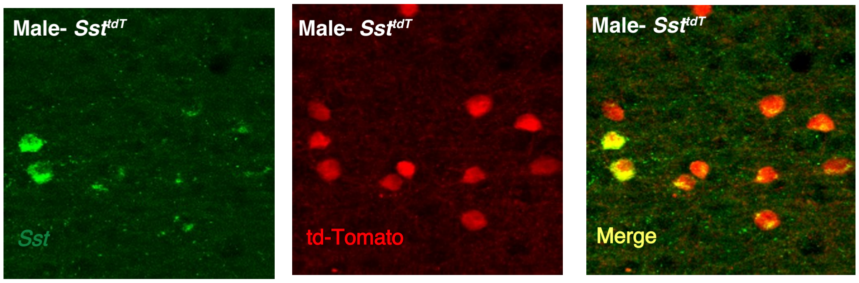

B

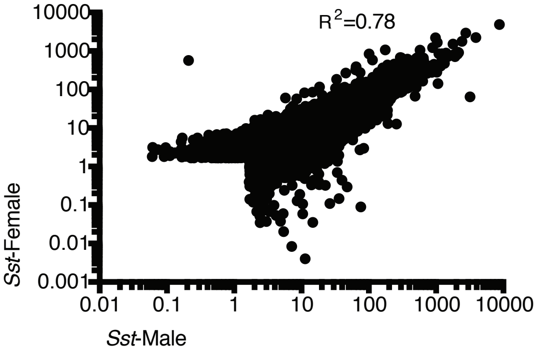

C

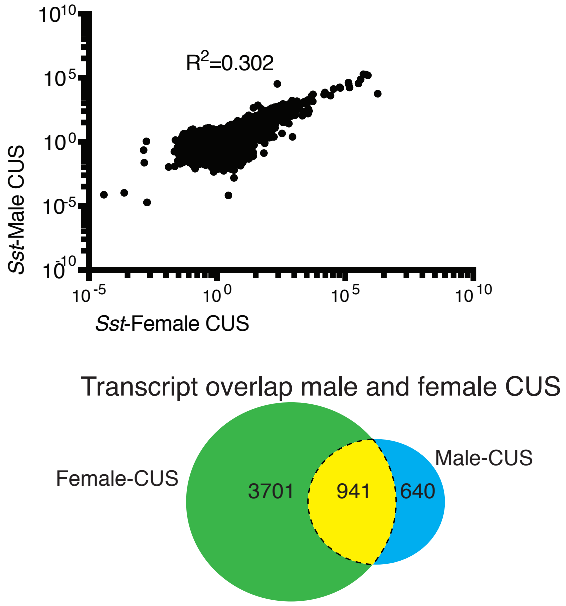

Figure S2

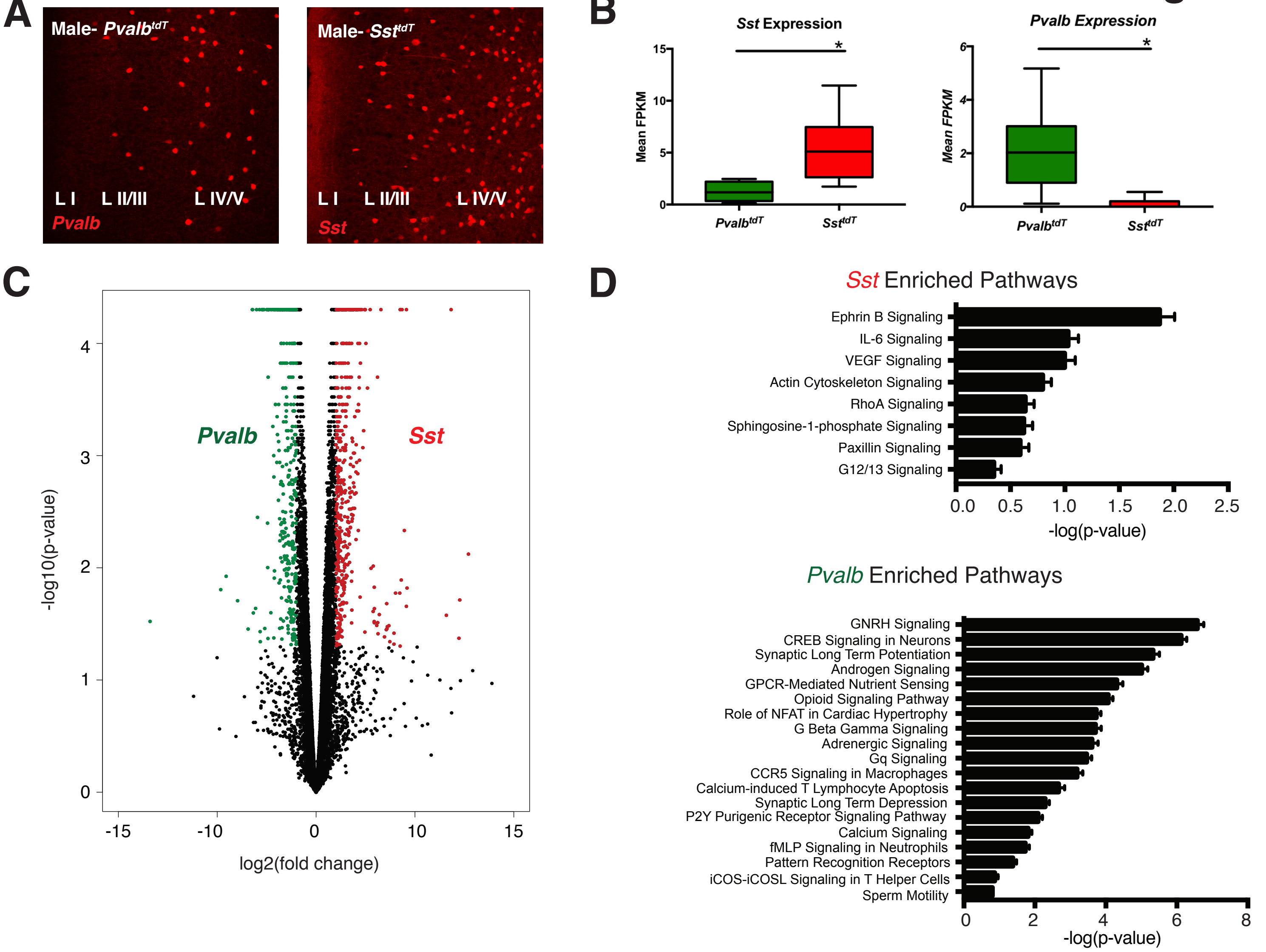
A
Male- Pvalb^tdT
L I
L II/III
L IV/V
Pvalb
Male- Sst^tdT
L I
L II/III
L IV/V
Sst
B
Sst Expression
Mean FPKM
Pvalb^tdT
Sst^tdT
*
Pvalb Expression
Mean FPKM
Pvalb^tdT
Sst^tdT
*
C
Pvalb
Sst
-log10(p-value)
log2(fold change)
D
Sst Enriched Pathways
Ephrin B Signaling
IL-6 Signaling
VEGF Signaling
Actin Cytoskeleton Signaling
RhoA Signaling
Sphingosine-1-phosphate Signaling
Paxillin Signaling
G12/13 Signaling
-log(p-value)
Pvalb Enriched Pathways
GNRH Signaling
CREB Signaling in Neurons
Synaptic Long Term Potentiation
Androgen Signaling
GPCR-Mediated Nutrient Sensing
Opioid Signaling Pathway
Role of NFAT in Cardiac Hypertrophy
G Beta Gamma Signaling
Adrenergic Signaling
Gq Signaling
CCR5 Signaling in Macrophages
Calcium-induced T Lymphocyte Apoptosis
Synaptic Long Term Depression
P2Y Purigenic Receptor Signaling Pathway
Calcium Signaling
fMLP Signaling in Neutrophils
Pattern Recognition Receptors
iCOS-iCOSL Signaling in T Helper Cells
Sperm Motility
-log(p-value)

Figure S3

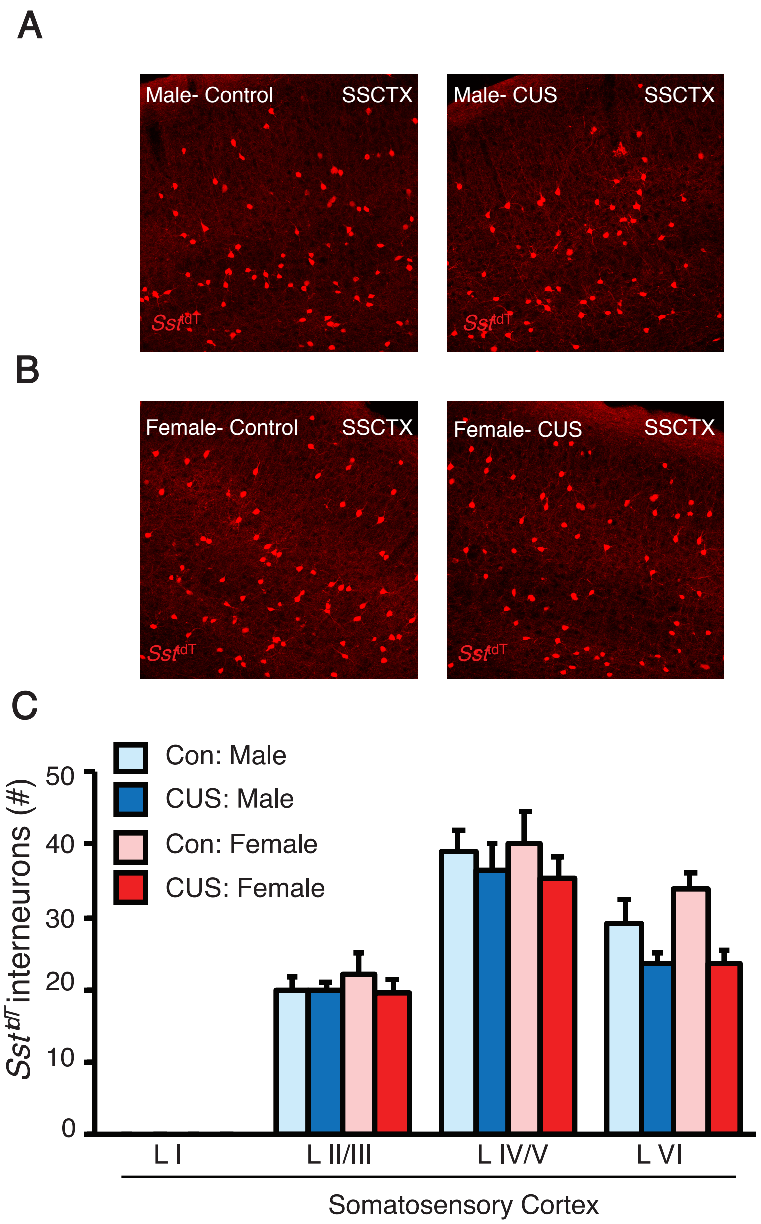
A
Male- Control
SSCTX
SsttdT
Male- CUS
SSCTX
SsttdT
B
Female- Control
SSCTX
SsttdT
Female- CUS
SSCTX
SsttdT
C
Con: Male
CUS: Male
Con: Female
CUS: Female
SsttdT interneurons (#)
0
10
20
30
40
50
L I
L II/III
L IV/V
L VI
Somatosensory Cortex

Figure S4

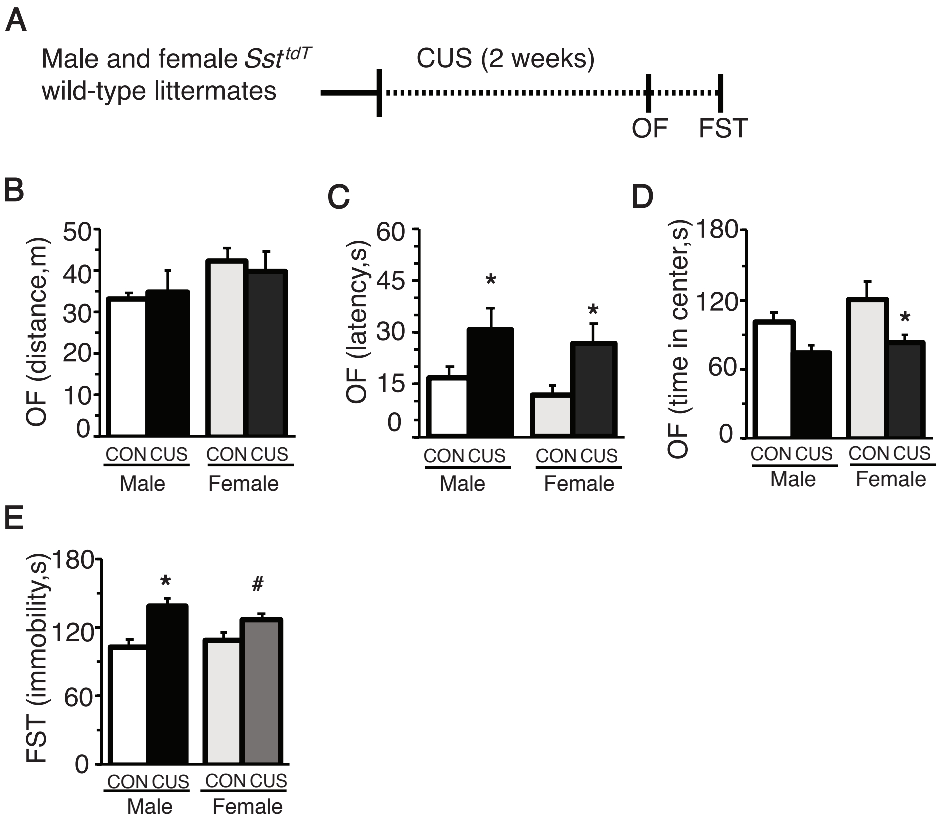

A
Male and female Sst^{tdT} wild-type littermates
CUS (2 weeks)
OF
FST
B
OF (distance,m)
50
40
30
20
10
0
CON CUS
Male
CON CUS
Female
C
OF (latency,s)
60
45
30
15
0
*
*
CON CUS
Male
CON CUS
Female
D
OF (time in center,s)
180
120
60
0
*
CON CUS
Male
CON CUS
Female
E
FST (immobility,s)
180
120
60
0
*
#
CON CUS
Male
CON CUS
Female

Figure S5

A

Pathways enriched for DEGs between baseline human males and females

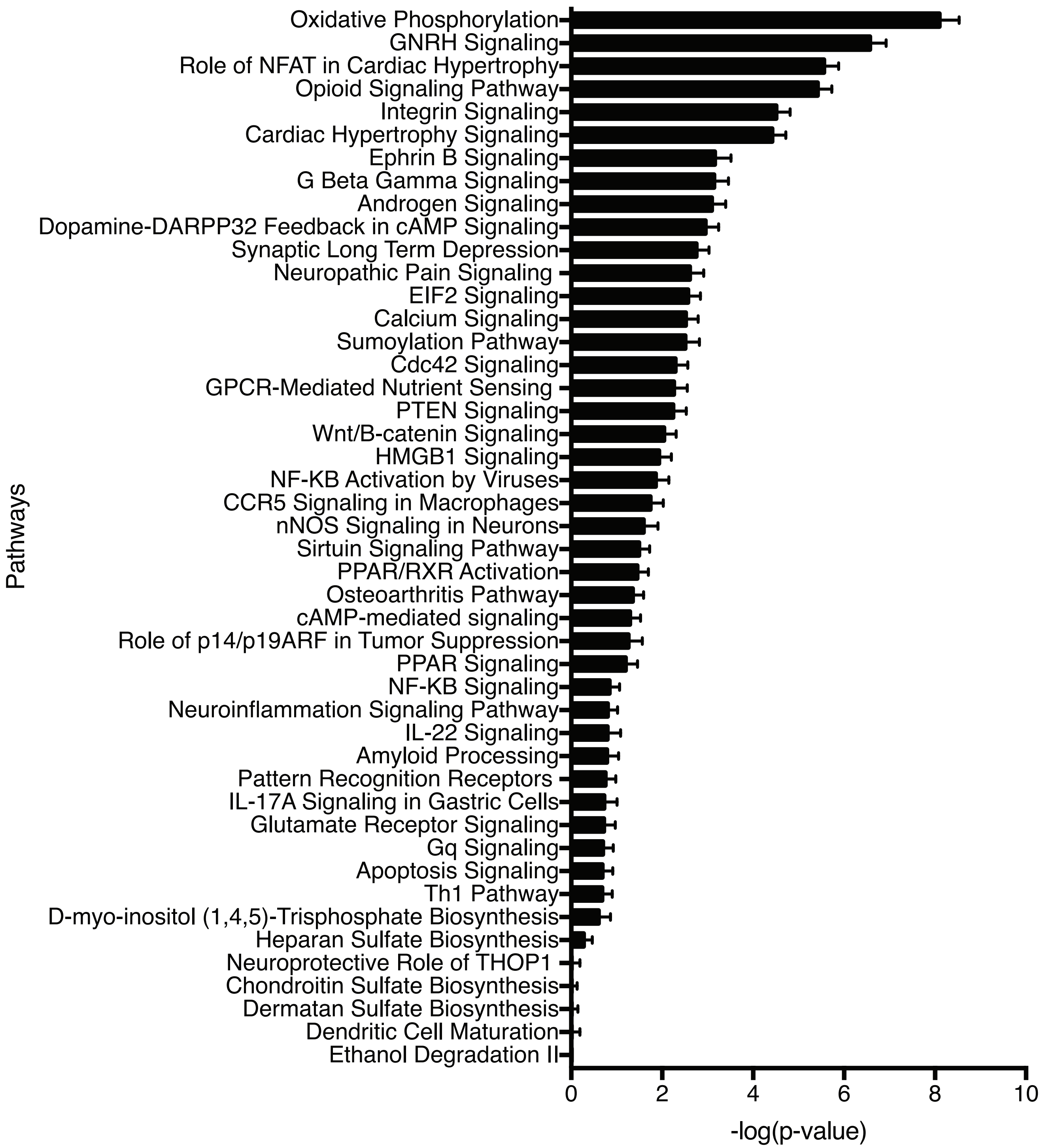

Supplement: Supplementary file 1 — Supplemental Figures [file 41398_2019_642_MOESM1_ESM.pdf]
