# Supplementary material for: Prefrontal cortex interneurons display dynamic sex-specific stress-induced transcriptomes
Source: Transl Psychiatry. 2019 Nov 11;9:292. doi: 10.1038/s41398-019-0642-z (PMC6848179; doi:10.1038/s41398-019-0642-z)
Supplement: Supplementary file 6 — Supplemental legends [file 41398_2019_642_MOESM6_ESM.docx]

**Supplemental File Legends**

**Supplemental Figure 1: Global and differentially expressed correlations between baseline male and female** *Sst^tdT^* **interneurons and after CUS. (A)** Representative micrographs of mPFC sections stained for endogenous Sst (green), *Sst^tdT^* (red), and merged (yellow) to show overlap. **(B)** Correlation of global transcript levels between male and female *Sst^tdT^* neurons. Pearson coefficient was calculated for correlation (R^2^=0.78). **(C)** Correlation of global transcript levels between CUS males and CUS female *Sst^tdT^* interneurons. Pearson coefficient was calculated for correlation (R^2^=0.302). **(C, lower)** 941transcripts overlapped between CUS female DEGs and male CUS DEGs. 336 transcripts were up regulated. 293 overlapped in direction while 43 did not. 605 transcripts were down regulated. 601 overlapped while 4 did not.

**Supplemental Figure 2: Interneuron subtype-specific transcriptional profiles reveal enrichment of pathways involved in synaptic potentiation and cytoskeletal signaling. (A)** Micrographs showing *Sst^tdT^* and *Pvalb^tdT^* labeled interneurons in the infralimbic PFC of mice and schematic of interneuron subtype isolation and RNA sequencing. **(B)** Box plots showing higher FPKM of *Pvalb* transcripts from *Pvalb^tdT^* sorted cells and higher FPKM *Sst* transcripts from *Sst^tdT^* sorted cells. *P<0.05, Mann-Whitney U test. **(C)** Volcano plot of differential gene expression between *Sst^tdT^* and *Pvalb^tdT^* interneurons, n=8 *Sst^tdT^* or *Pvalb^tdT^* mice (FDR<0.05). Transcripts enriched in *Sst^tdT^* interneurons are red and those enriched in *Pvalb^tdT^*are green. There were 1481 transcripts that significantly differed between *Sst^tdT^* and *Pvalb^tdT^* sorted cells. 156 genes were expressed in *Pvalb^tdT^* that were not expressed in *Sst^tdT^* cells and 228 were exclusive to *Sst^tdT^*. **(D)** Pathway analysis of transcripts enriched in either subtype. Pathways are rank ordered by z-score value.

**Supplemental Figure 3. CUS does not alter the number of *Sst^tdT^*** **interneurons in somatosensory cortex. (A)** Male and female *Sst^tdT^* mice were exposed to 14 days of CUS or left undisturbed as controls and brains were collected for immunohistology. Representative confocal images of *Sst^tdT^* interneurons in the somatosensory cortex of male or female mice. **(B)** Quantification of the total number of *Sst^tdT^* interneurons in each lamina of the somatosensory cortex (*n* = 3/ group). Overall CUS did not affect the number of *Sst^tdT^* interneurons in the somatosensory cortex.

**Supplemental Figure 4. CUS causes depressive-like behaviors in *Sst^tdT^*** **mice.** **(A)** Schedule of CUS paradigm and behavioral testing. **(B)** Total distance in the open field (main effect of sex F_1,28_=9.20 p<0.005); **(C)** latency (main effect of stress F_1,28_=8.42, p<0.007); and **(D)** time spent in the center of the field (main effect of stress F_1,28_=9.05, p<0.005) are shown. **(E)** Time spent immobile in the forced swim test (main effect of stress F_1,28_=18.62, p<0.0001). Bars represent mean +/- SEM, n=8 per group. *P<0.05, #P=0.08, significantly different from respective control group-based ANOVA.

**Supplemental Figure 5**. Complete list of enriched pathways of transcripts differentially expressed between human males and females in area 25.

**Supplemental Table 1**. All significant DEGs from all RNA-seq experiments.

**Supplemental Table 2**. List of transcripts overlapping between control female DEGs and male CUS DEGs.

**Supplemental Table 3**. List of transcripts overlapping between male CUS and female CUS DEGs.

**Supplemental Table 4**. List of transcripts overlapping between human male MDD DEGs and control female DEGs.
